# Optimizing process-based models to predict current and future soil organic carbon stocks at high-resolution

**DOI:** 10.1038/s41598-022-14224-8

**Published:** 2022-06-25

**Authors:** Derek Pierson, Kathleen A. Lohse, William R. Wieder, Nicholas R. Patton, Jeremy Facer, Marie-Anne de Graaff, Katerina Georgiou, Mark S. Seyfried, Gerald Flerchinger, Ryan Will

**Affiliations:** 1 Department of Biological Sciences, Idaho State University, Pocatello, ID USA; 2 Department of Geosciences, Idaho State University, Pocatello, ID USA; 3 Climate and Global Dynamics Laboratory, National Center for Atmospheric Research, Boulder, CO USA; 4 Institute of Arctic and Alpine Research, University of Colorado, Boulder, CO USA; 5 School of Earth and Environment, University of Canterbury, Christchurch, New Zealand; 6 Department of Biological Sciences, Boise State University, Boise, ID USA; 7 Physical and Life Sciences Directorate, Lawrence Livermore National Laboratory, Livermore, CA USA; 8 Agricultural Research Service, Northwest Watershed Research Center, Boise, ID USA; 9 Department of Geosciences, Boise State University, Boise, ID USA

**Keywords:** Carbon cycle, Carbon cycle, Environmental impact

## Abstract

From hillslope to small catchment scales (< 50 km^2^), soil carbon management and mitigation policies rely on estimates and projections of soil organic carbon (SOC) stocks. Here we apply a process-based modeling approach that parameterizes the MIcrobial-MIneral Carbon Stabilization (MIMICS) model with SOC measurements and remotely sensed environmental data from the Reynolds Creek Experimental Watershed in SW Idaho, USA. Calibrating model parameters reduced error between simulated and observed SOC stocks by 25%, relative to the initial parameter estimates and better captured local gradients in climate and productivity. The calibrated parameter ensemble was used to produce spatially continuous, high-resolution (10 m^2^) estimates of stocks and associated uncertainties of litter, microbial biomass, particulate, and protected SOC pools across the complex landscape. Subsequent projections of SOC response to idealized environmental disturbances illustrate the spatial complexity of potential SOC vulnerabilities across the watershed. Parametric uncertainty generated physicochemically protected soil C stocks that varied by a mean factor of 4.4 × across individual locations in the watershed and a − 14.9 to + 20.4% range in potential SOC stock response to idealized disturbances, illustrating the need for additional measurements of soil carbon fractions and their turnover time to improve confidence in the MIMICS simulations of SOC dynamics.

## Introduction

A large portion of the world’s actively cycled carbon (C) is stored in surface soils^1,2^. With changes in global climate and land use accelerating at unprecedented rates^3–5^, there is an emergent need to project soil organic carbon (SOC) dynamics under changing environmental conditions. A growing body of work focuses on potential global-scale SOC responses to climate change^6–8^, but simulating soil carbon dynamics at more localized scales (hillslope and small watershed catchment, < 50 km^2^) offers opportunities to parameterize process-based models at resolutions that have a greater relevance to policy and management^9,10^. This more local approach also better connects models and field observations, allowing stronger inference of the proximal controls over SOC persistence and turnover that ultimately builds confidence in model projections^11,12^. Such advances, however, require quantitative tools that can be used to link observations and models to calibrate model parameters, assess parametric uncertainty, and generate high resolution estimates of soil carbon stocks and potential vulnerabilities.

Current methodologies for estimating soil carbon fall into two main categories, digital soil mapping and process-based modeling. Digital soil mapping uses statistical correlations between climate, vegetation, topography, soil properties, and soil C stocks to generate high resolution estimates of SOC stocks^13–16^. While often accurate for predicting SOC stocks at fine scales^17^, digital mapping methods generally have limited ability to project future SOC dynamics and responses since the statistical estimation of SOC does not adequately derive how biogeochemical interactions and environmental conditions combine to govern SOC stocks^18^. In contrast, process-based models have been extensively relied upon for projecting regional to global scale SOC stocks and their vulnerabilities to changing conditions^19–22^. Process-based models mathematically approximate the processes through which soil C enters the soil, decomposes, and then is either stored in soil or cycled back to the atmosphere. The use of process-based models for projecting SOC dynamics at high spatial resolution (e.g., < 250 m^2^) is predicated by the availability of accurate, high-resolution forcing data for model calibration and the extrapolation of projections across complex landscapes. Rapid advances in monitoring technology, remote sensing, and machine learning present novel opportunities to generate such environmental data^23^. In addition to these technical challenges, major uncertainties also exist regarding the viability of using model parameters that were calibrated for regional- to global-scale simulations for projections at much smaller, hillslope- to watershed-scales.

Controls on SOC stocks are currently thought to change with the scale of observation and inference^24,25^. Global-scale simulations typically operate at a coarse spatial resolution (e.g. > 50 km^2^). At these scales, process-based models of SOC have produced accurate estimates of SOC stocks based predominantly on the influences of soil temperature and moisture, net primary productivity and soil texture^26–29^. At smaller scales, additional environmental controls are often inferred to have more pronounced effects on SOC stocks across complex landscapes, including controls imposed by detrital input quality^30^, microbial community composition^31^ and physical factors such as landscape position^16^ and erosion^32,33^. These additional influences on SOC dynamics often drive local heterogeneity in SOC stocks. Indeed, the importance of including such local biogeochemical controls in process model representations continues to be demonstrated and theorized^22,34^, leading many modern process-based models to incorporate components relating to litter chemistry and microbial processing^26,27,35^. These advances in the process-based SOC model constructs are likely to improve the potential for fine scale process model applications.

**Figure 1:**
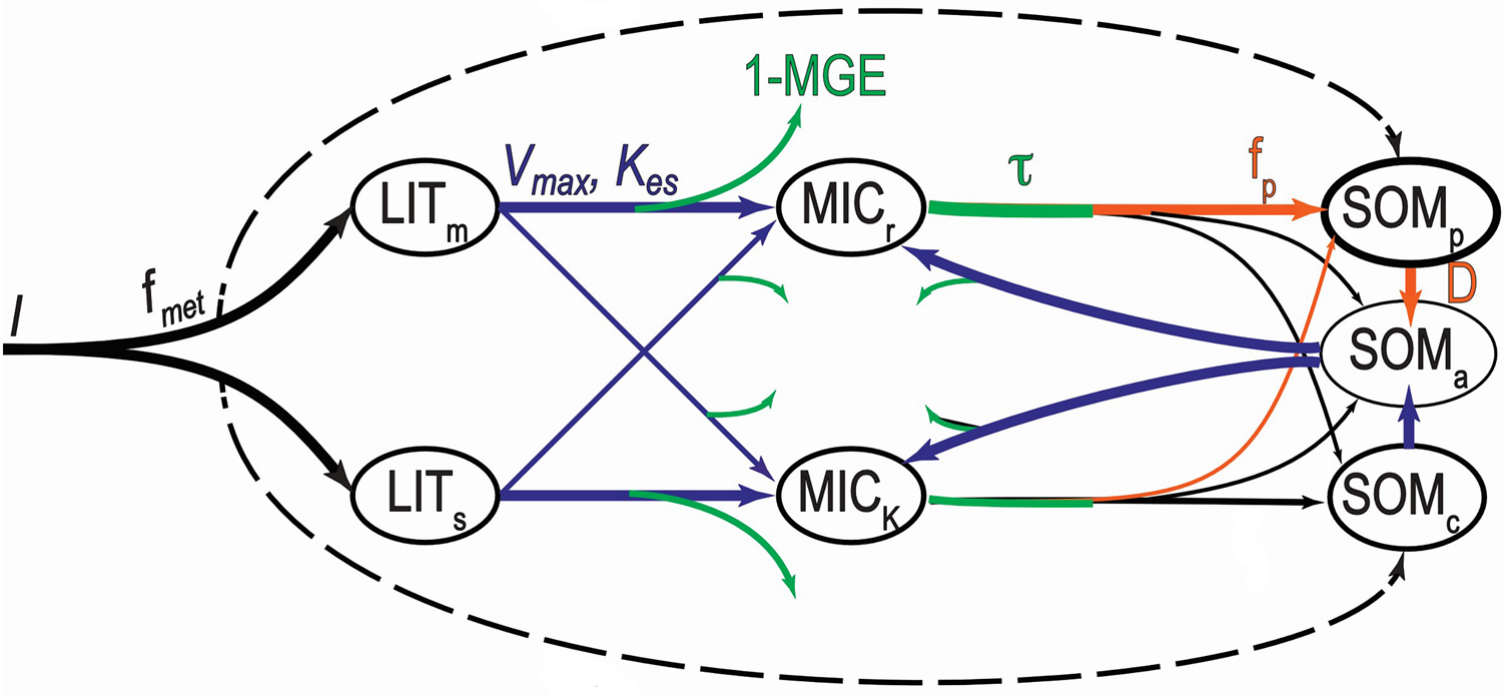
Diagram of the MIcrobial-MIneral Carbon Stabilization (MIMICS) model, which explicitly considers microbial functional diversity by simulating two functional groups (MIC_r_, inefficient, fast-growers; MIC_K_, conservative, slow-growers) and their potential effects on litter decomposition and soil organic matter persistence (available SOM_a_; chemically protected SOM_c_; physically protected SOM_p_). Litter C inputs are initially partitioned (f_met_) between litter pools (structural LIT_s_; metabolic LIT_m_), while a lesser fraction (f_i_) transfers directly to the SOM pools. Parameters used for model calibration and validation related to microbial catabolic capacity (V_max_ and K_es_, blue lines), microbial anabolism (MGE and τ, green lines), and physicochemical protection of SOM (f_p_ and D, orange lines). See also Table 1.

Here we develop a method for model parameter optimization and high-resolution mapping of simulated SOC stocks across complex landscapes at spatial scales consistent with field observations and land management practices. This work combines the MIcrobial-MIneral Carbon Stabilization model (MIMICS; Fig. 1)^22,26^ with high-resolution input data, soil carbon measurements, and a Monte Carlo approach for parameter optimization and associated uncertainty analysis. We specifically explore the impact of parametric uncertainty on model projections of SOC stocks and their potential sensitivities across a complex landscape with strong climate gradients and diverse soil properties at the Reynolds Creek Experimental Watershed and Critical Zone Observatory (RCEW-CZO)^36^. This work demonstrates how a variety of measurements collected across environmental gradients at the pedon and watershed scale improves our understanding and prediction of soil carbon storage and processes at management relevant scales. Ultimately, results from this study advance the general ability to model, map and project the sensitivity of SOC stocks at fine spatial scales, thus providing an important pathway for scientists, land managers and policymakers to gain more detailed spatial information about the existing size and future stability of SOC stocks across complex landscapes.

## Results

### Parameter estimation and uncertainty quantification

**Figure 2:**
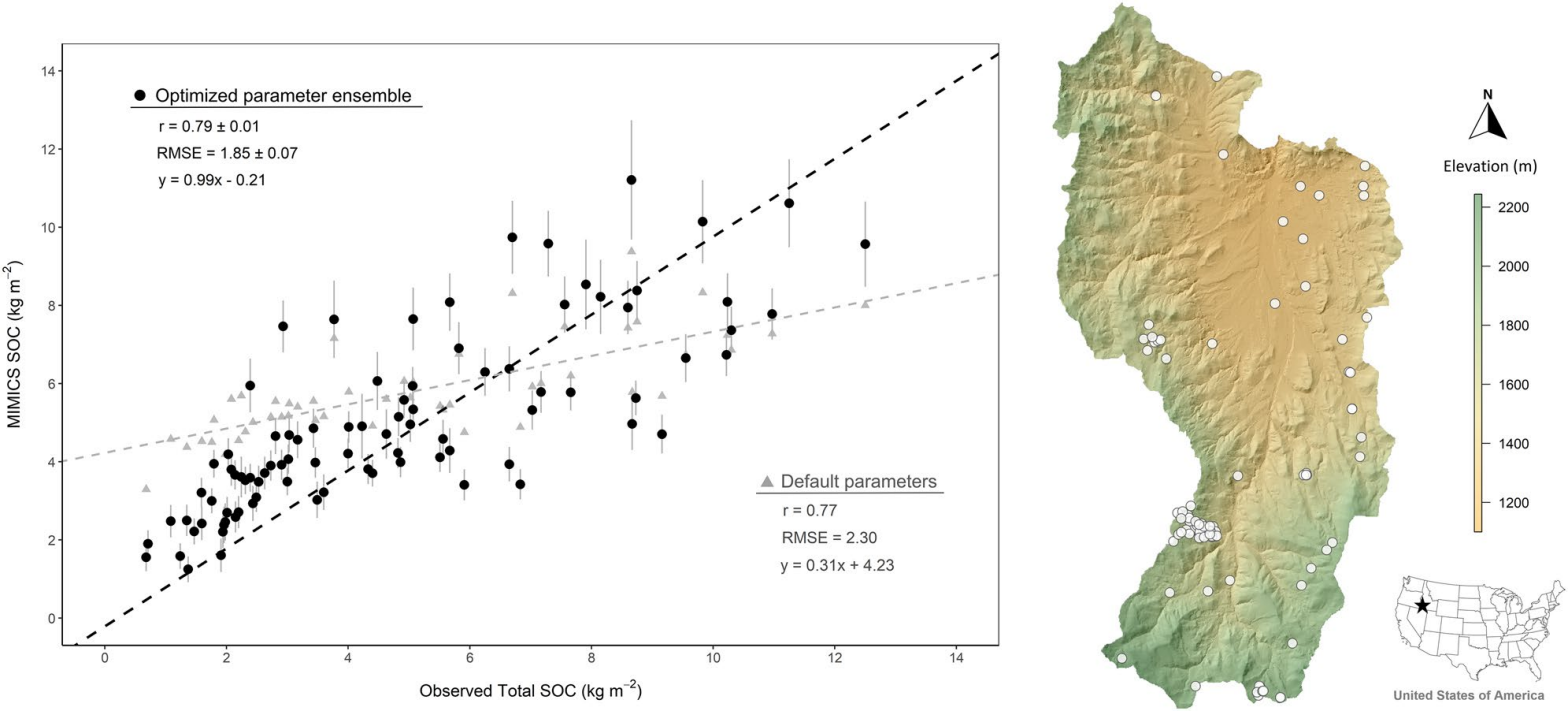
Comparison of the MIMICS model estimates with observed soil organic carbon stocks (kg C m^2^, 0–30 cm soil depth) at locations across the Reynolds Creek Experimental Watershed and Critical Zone Observatory (n = 89). Black dots and associated error bars denote the mean ± standard deviation of the model estimates produced using each member of the optimized parameter ensemble. Grey triangles represent MIMICS estimates using the default MIMICS model parameterization. (Generated by free software R, https://www.R-project.org/).

Initial simulations of the MIMICS model using high-resolution forcing data (Fig. SI1) and the parameters provided by Wieder et al.^26^ (i.e., priors) resulted in simulated SOC stocks that showed biases relative to field observations across the Reynolds Creek Critical Zone Observatory (Fig. 2). The initial default parameters underpredicted higher SOC stocks and overpredicted lower SOC stocks (Fig. 2; r = 0.77, RMSE = 2.30 kgC m^-2^). Optimizing the model parameters with a Markov Chain Monte Carlo (MCMC) based method revealed that many different parameter combinations were capable of producing similarly accurate estimates of SOC stocks across the Reynolds Creek Critical Zone Observatory (r = 0.77–0.82; root mean square error (RMSE) = 1.7–2.0 kgC m^-2^; Examples of differing MCMC solutions are shown in Fig. SI2). Thus, we generated an ensemble of unique parameter combinations where each parameterization yielded a RMSE < 2 kgC m^-2^ between the model estimates and field observations of SOC stocks across the Reynolds Creek Critical Zone Observatory (Fig. 2; n = 30 unique parameter combinations). Overall, the ensemble of optimized parameter combinations for MIMICS improved soil carbon predictions by eliminating bias observed when using the priors (Fig. 2) and reducing the estimate RMSE by as much as 0.58 kgC m^-2^ (25%).

**Figure 3:**
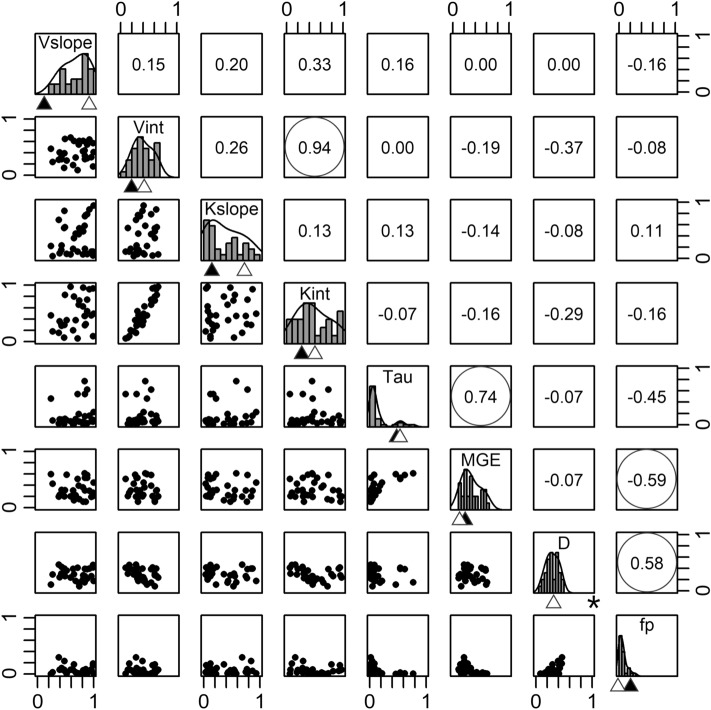
MIMICS model parameter space and relationships among parameters in the parameter ensemble that produced similarly accurate (RMSE 1.8–2.0) estimates of total soil organic C stocks across the Reynolds Creek Experimental Watershed and Critical Zone Observatory (n = 30 member parameter ensemble). Normalized parameter range coincides with the scaling factor proposal range for each parameter (see Table 1). Histograms on the diagonal represent the uncertainty in parameter estimates from the parameter ensemble. Triangles below histograms represent default (closed) and best-fit (open) parameter values. Off-diagonal panels display pairwise correlations and correlation coefficients between individual parameters, with statistically significant correlations circled (*p* < 0.05). *Default parameter line for D is beyond plot scale (D_default_ = 3.1). (Generated by free software R, https://www.R-project.org/).

The parameter space covered by our ensemble produced near equivalent estimates for total SOC stocks across the RCEW-CZO (see histograms in Fig. 3). The calibrated model consistently found parameterizations that show higher temperature sensitivity of microbial catabolic capacity (*V*_*slope*_*, V*_*int*_*, K*_*slope*_*, K*_*int*_), relative to the initial model parameterizations used by Wieder et al.^26^. Similarly, parameters related to physicochemical protection of soil C (f_p_ and D) are lower than the initial model parameterizations, reflecting the longer turnover time of physicochemically protected organic matter (SOM_p_) with our optimized parameter values. Finally, the parameter ensemble generated a normal distribution for parameter estimates of MGE and log-normal distributions for microbial turnover rates, however the ‘best’ parameter estimates for microbial anabolism were not dramatically different from initial parameter values.

Our parameter ensemble also revealed strong autocorrelations for particular parameter combinations in MIMICS. For example, the decomposition rate intercept terms in the model *(V*_*int*_ and *K*_*int*_) were positively correlated (r = 0.94, see scatter plots on Fig. 3). We also observed correlations between the parameters controlling the fate of carbon through microbial anabolism with each other and terms associated with the persistence of soil C in the physicochemical protected pool (SOM_p_, Fig. 1). Specifically, we observed that parameter estimates for microbial growth efficiency (*MGE*) were positively correlated with microbial turnover rates (*τ*, r = 0.74; Fig. 3) and negatively correlated with the fraction of SOM partitioned to the protected SOM pool (*f*_*p*_, r = -0.59). Further, *f*_*p*_ was positively correlated with desorption (*D*) of SOM_p_ to the available SOM pool (r = 0.58). No correlations (r > 0.4) were observed between the decomposition rate parameters associated with microbial catabolic capacity and those related to microbial anabolism or persistence of soil C in physicochemical protected pools.

**Figure 4:**
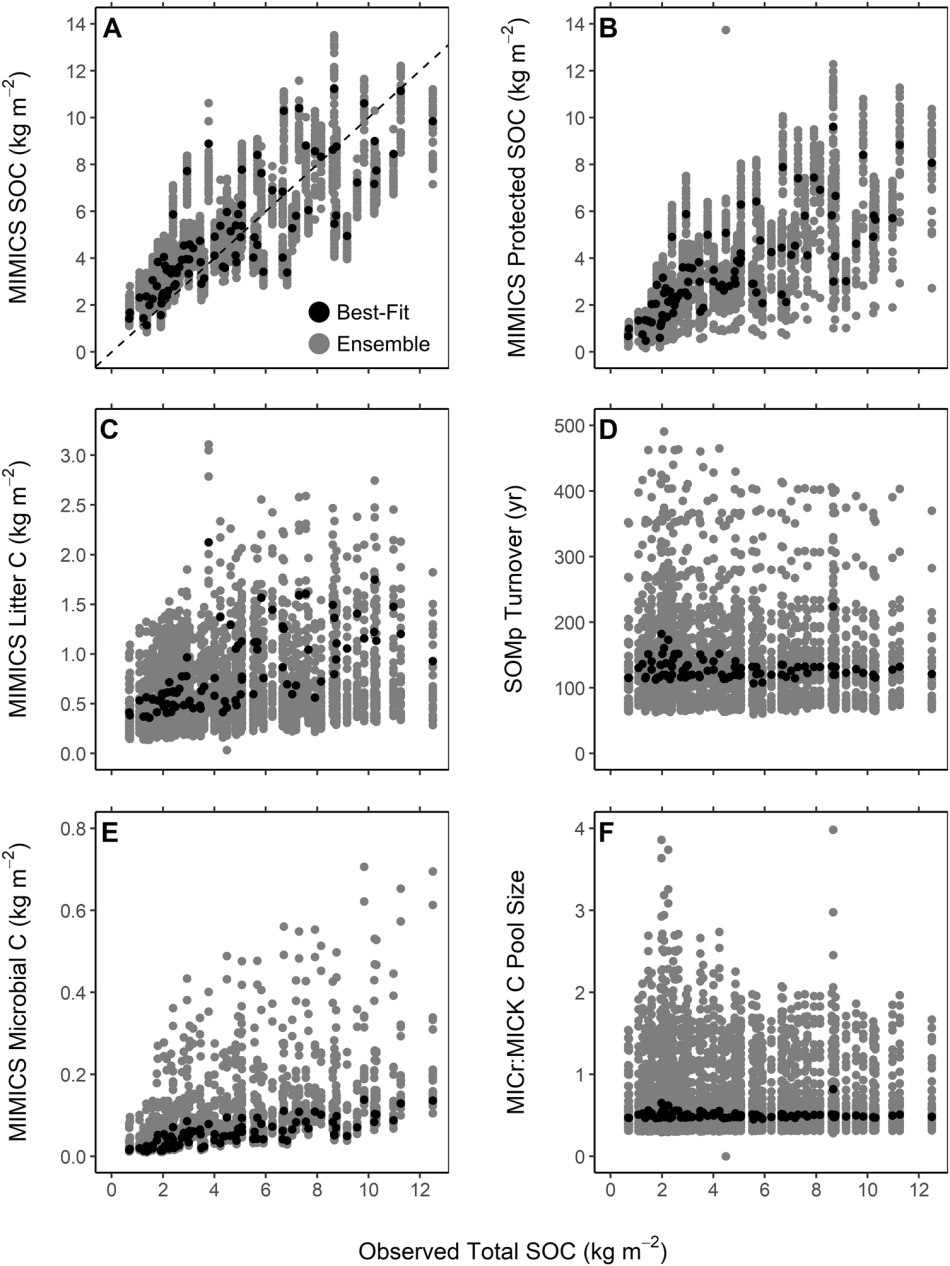
MIMICS estimate variability relative to observed SOC stocks for the best (lowest RMSE) MC determined parameterization and a larger ensemble of MIMICS model parameterizations (n = 30) with SOC stock estimate RMSE < 2. (Generated by free software R, https://www.R-project.org/).

While all parameterizations in the parameter ensemble resulted in similarly robust estimates of total SOC stocks (RMSE < 2.0 kg C m^−2^; Fig. 4A), the parametric uncertainty across the ensemble (i.e. the range of parameter combinations) led to greater variability in the model estimates of underlying litter, microbial biomass, and soil C pools than for estimates of total SOC stocks (Fig. 4). Across the parameter ensemble, the protected soil carbon stocks (SOM_p_) simulated by MIMICS varied by a factor of 4.4 ± 2.0 at individual locations (mean ± 1*σ*; Fig. 4B). This variability in SOM_p_ pool sizes can in part be explained by uncertainty in the parameterization of the pool’s turnover time, which ranged from 75 to over 400 years (Fig. 4D). Similarly, across individual locations, litter C pool estimates varied by a factor of 6.9 ± 3.3 and the proportion of total SOC made up of microbial biomass varied by a factor of 7.7 ± 1.7 (Fig. 4C,E, respectively). Finally, the parametric uncertainty led to a large range in the relative abundance of copiotrophs (MIC_r_) and oligotrophs (MIC_K_) that were simulated across the RCEW-CZO, with a mean MIC_r_:MIC_K_ ratio of 0.80 ± 0.48 across individual locations (Fig. 4F).

### High-resolution mapping and sensitivities of SOC stocks

**Figure 5:**
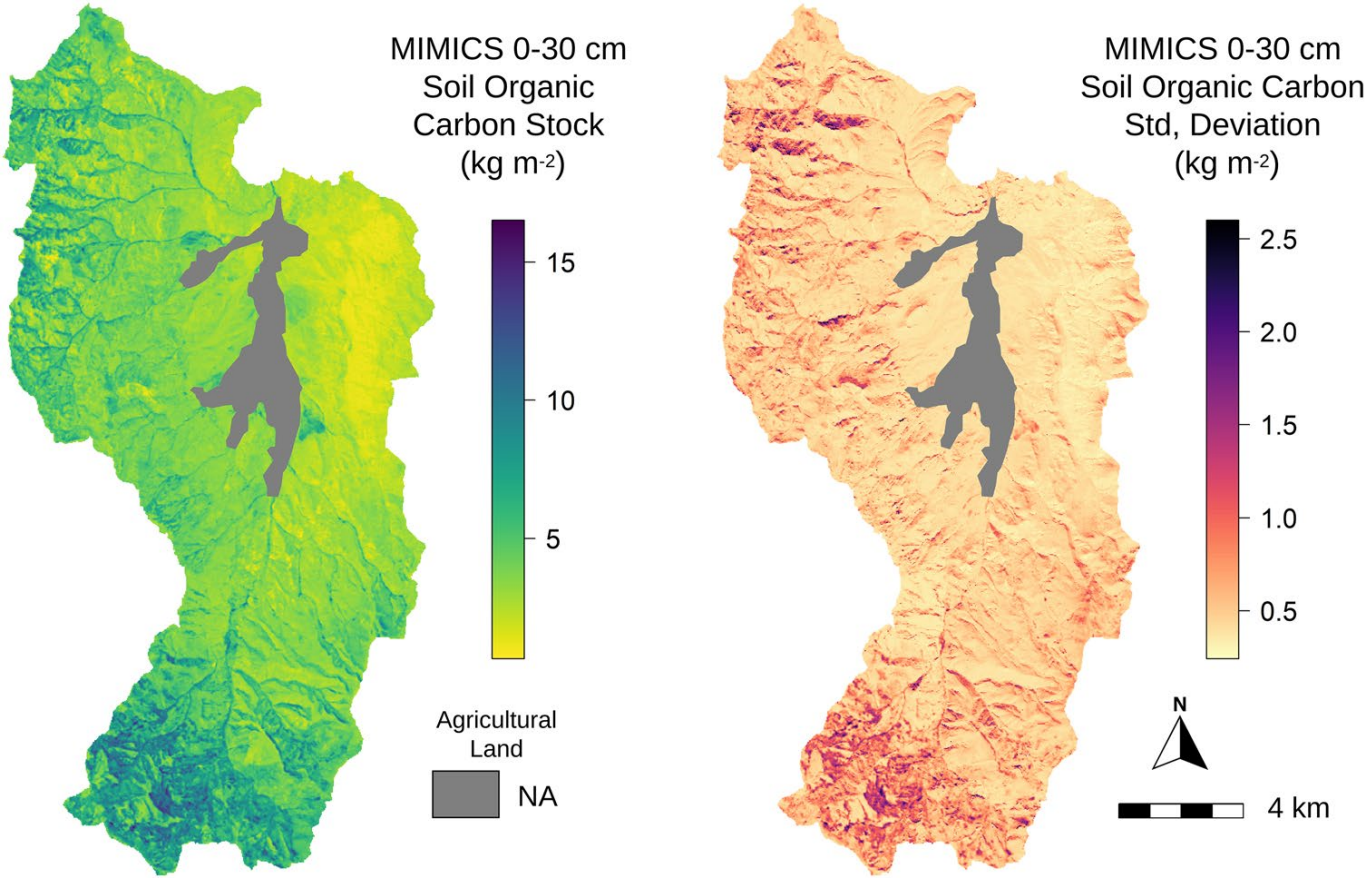
Ensemble (**A**) mean and (**B**) standard deviation of total soil organic carbon stocks simulated by the MIMICS model across the natural area of the Reynolds Creek Experimental Watershed and Critical Zone Observatory from 0–30 cm soil depth (n = 30 maps of soil C generated by the parameter ensemble produced from model calibration with site observations; Fig. 3a). (Generated by free software R, https://www.R-project.org/).

A spatially continuous map of estimated SOC stocks from 0 to 30 cm soil depth was generated for the RCEW-CZO by combining results from the parameter ensemble with gridded spatial data (*i.e.*, rasters; Fig. SI1) of the required model inputs (Fig. 5). We note that these rasters were also used to generate the forcing data used for model parameter optimization (above) based on field observations of SOC stocks. To generate a high-resolution map of 0–30 cm SOC stocks for the entire natural area of the RCEW-CZO, we calculated the steady-state carbon pools simulated by MIMICS at 2.4 million points evenly distributed across the watershed (10 m resolution). We repeated this with each of the 30 members of the parameter ensemble to calculate uncertainty estimates in the spatial distribution of belowground C stocks. Figure 5 shows the ensemble mean and associated parametric uncertainty (± 1*σ*) of soil C stocks across the watershed.

The high-resolution mapping with MIMICS shows that soil C stocks follow elevational trends at RCEW-CZO, while also reflecting more localized topographical differences in precipitation and mean annual temperature that drive shifts in vegetation communities, productivity, and soil development. Total SOC stocks are largest in the southern and western portion of the watershed, where higher elevations bring cooler temperatures and higher water availability, which supports more productive vegetation communities. Within elevational bands we also see strong influences of aspect on SOC stocks. On average across the watershed, soils on north-facing slopes hold 28% more SOC relative to south-facing slopes. For areas above 1600 m elevation, the aspect effects on SOC storage increase, with 41% more SOC stored on north-facing slopes relative to south-facing slopes. The largest uncertainties in soil C stocks correspond to areas with higher productivity and larger SOC stock estimates. Finally, areas where the dominant litter source shifts from grass and shrub species to tree species with relatively poor litter quality (higher lignin:N) also show greater total SOC uncertainty.

**Figure 6:**
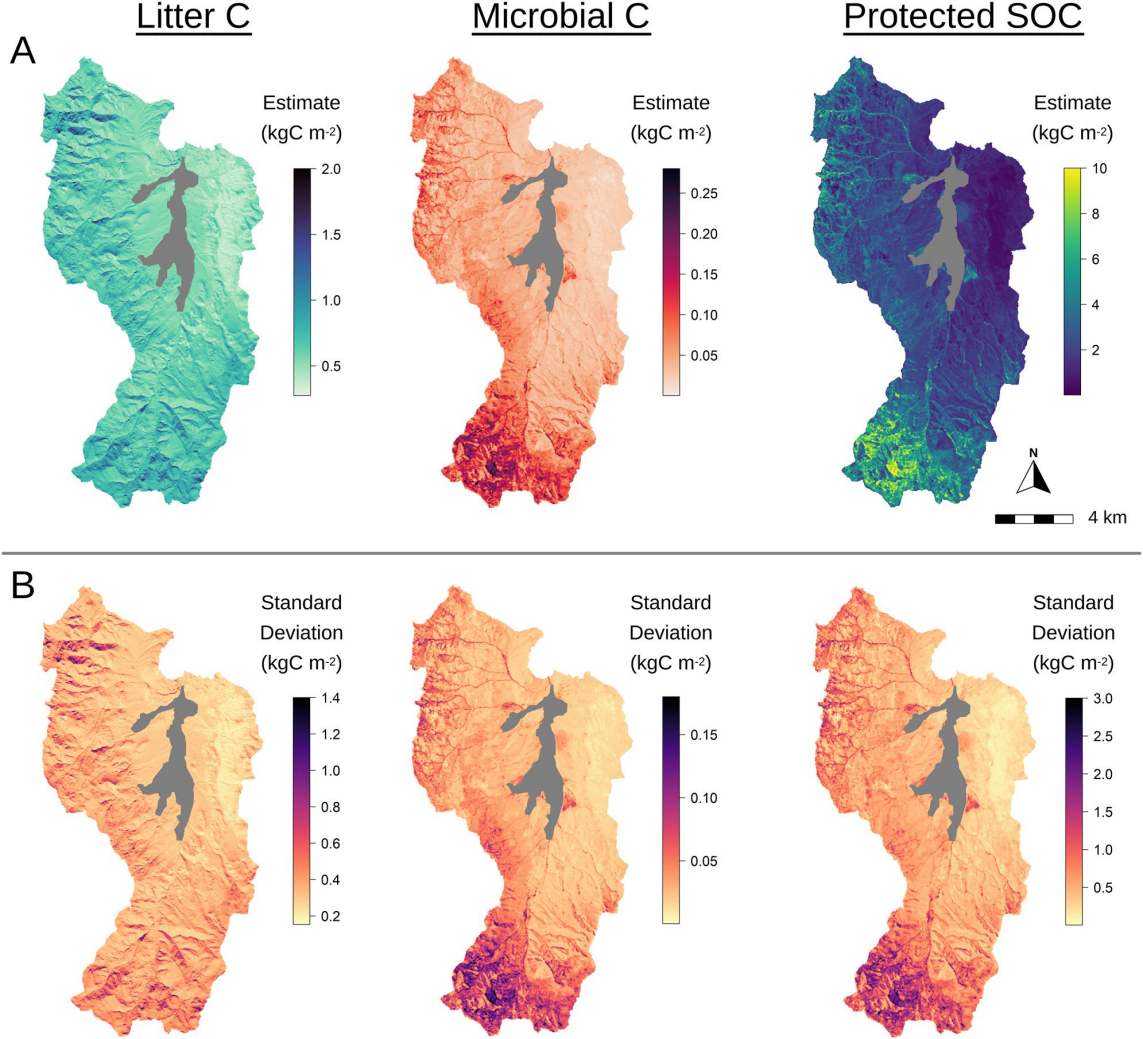
Ensemble (**A**) mean and (**B**) standard deviation of soil carbon pool stocks for 0–30 cm soil depth across the natural area of the Reynolds Creek Experimental Watershed and Critical Zone Observatory (n = 30 maps of soil C generated by the parameter ensemble produced from model calibration with site observations; Fig. 3a). (Generated by free software R, https://www.R-project.org/).

Our simulations also generate spatially explicit estimates of individual soil C pools including mean litter C, microbial C and protected SOC stocks and their associated uncertainty from the parameter ensemble (Fig. 6). Litter C pools strongly reflect the aspect effects across Reynolds Creek, with 59% more C stored in litter pools on northern facing slopes relative to south-facing slopes, reflecting greater control by soil temperature on litter C pools. We estimate that litter C pools comprise 14 ± 7% of the total SOC stock from 0 to 30 cm across the natural areas of Reynolds Creek. Microbial C stocks largely reflect spatial patterns in vegetative productivity (Fig. 6A, Fig. SI1A). We estimate protected SOM accounts for 67 ± 16% of the total SOC stock at Reynolds Creek, which also reflects spatial distributions of NPP estimates and less dependence on soil clay content, temperature or litter quality.

**Figure 7:**
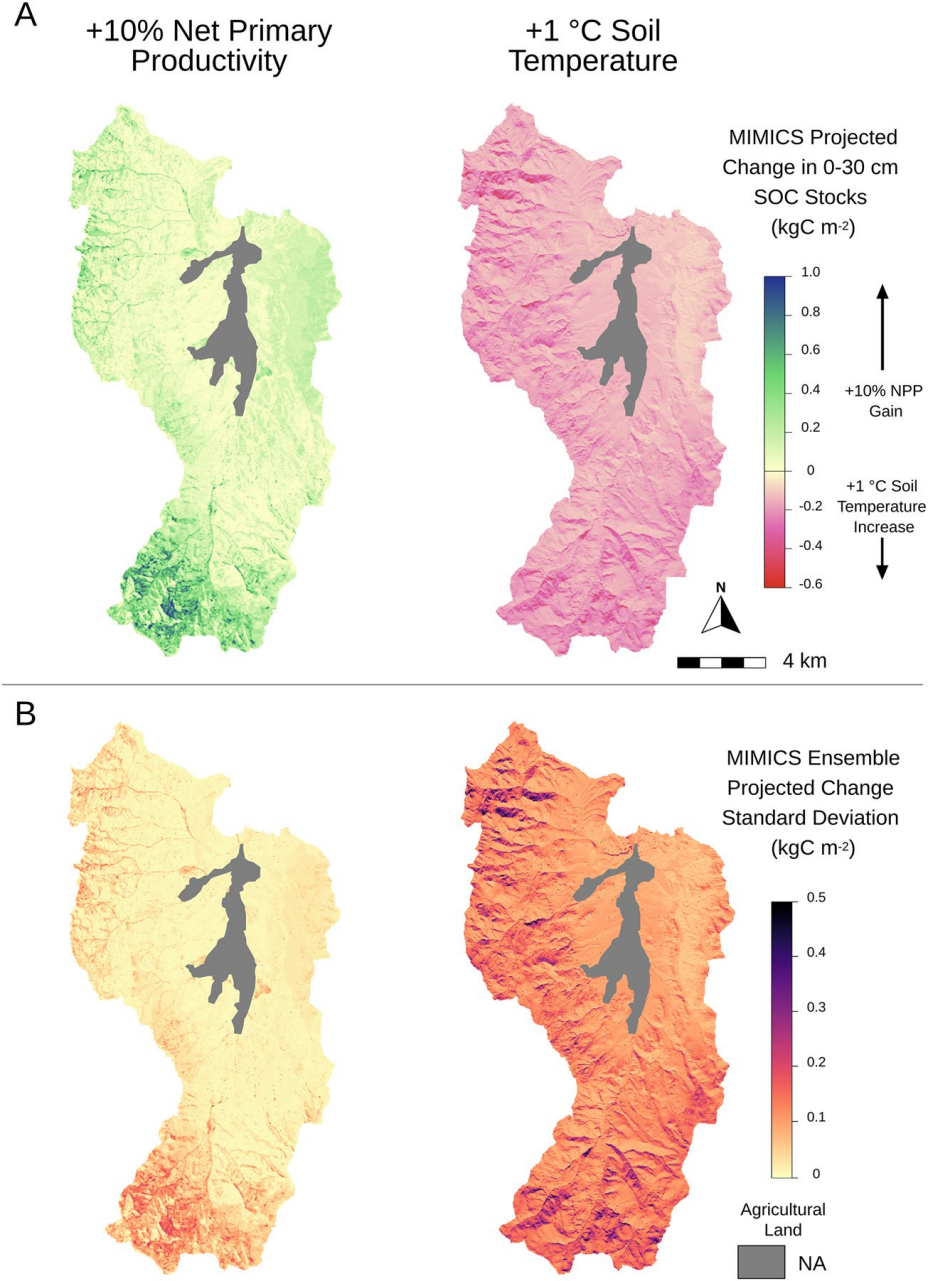
MIMICS projected (**A**) total change and (**B**) parametric uncertainty for change in 0–30 cm soil carbon stocks from 10% increase in net primary productivity (NPP) or 1 °C increase in mean annual soil temperature. (Generated by free software R, https://www.R-project.org/).

To project the sensitivity of existing soil C stocks to idealized environmental perturbations, we used the parameter ensemble to estimate soil C stock response to a uniform 10% increase in NPP and a 1 °C increase in mean annual soil temperature (Fig. 7). Gains in soil C from the increase in NPP were far greater in the southern, high elevation regions of the watershed where NPP is relatively high. The simulated 10% increase in NPP led to a total gain of 3.6 ± 0.6 GgC from 0–30 cm soil depth across Reynolds Creek, an increase of 3.5 ± 0.6% relative to initial stocks. Across ensemble member simulations, the mean estimated increase in SOC stocks across the watershed from the 10% increase in GPP ranged from 1.7—20.4% of existing stocks. In contrast, the 1 °C increase in soil temperature led to a more spatially consistent loss of soil C across Reynolds Creek, resulting in an estimated loss of 3.9 ± 2.8 GgC from 0–30 cm soil depth, or -3.7 ± 2.8% of initial stocks. Overall, projection uncertainty was much greater for the simulated change in soil temperature relative to the applied change in NPP (Fig. 7B). However, the range in net effect on SOC stocks was slightly smaller across the parameter ensemble. Across all ensemble simulations, the mean estimated change in SOC stocks from the 1 °C increase in soil temperature ranged from -1.1 to -14.9% of existing stocks.

## Discussion

### Overview

By coupling fine-resolution forcing data with model parameter optimization, we produced high resolution (10 m^2^) estimates of soil carbon stocks across the natural land area of the RCEW-CZO (239 km^2^ of complex terrain; Fig. 5). The use of high-resolution forcing data proved suitable for model performance when combined with parameter optimization, as the correlation between model projections and field observations of SOC stocks aligned with previous MIMICS applications (Fig. 2)^26^ and studies involving digital soil mapping methods^17^. Optimization of the model parameters was critical for reducing estimate systematic bias from the priors used in the initial simulations and reducing the RMSE between simulated and observed SOC stocks (Fig. 2). These results directly demonstrate the viability of combining process-based modelling and remotely sensed data products for simulating SOC stocks across complex landscapes.

Similar accurate, high-resolution estimates of SOC stocks across a complex environmental landscape have, to our knowledge, not been achieved previously using a process-based model such as MIMICS. In contrast to similar scale estimates of SOC stocks produced by statistical methods^16,17^, our process-based modeling approach simulates the controls on the decomposition, accumulation, and loss of SOC, providing predictive capacity to project soil C responses to changing environmental conditions. Perhaps the most similar study to date was conducted by Lu et al.^37^, who used spatial forcing data with a process-based model to project SOC stocks at 250 m^2^ resolution (r = 0.64) for a 7700 km^2^ watershed in the Loess Plateau, China. Our ability to project SOC stocks for the RCEW-CZO at a 10 m^2^ resolution with high accuracy (r = 0.79, Fig. 2) is a combined product of recent advances in remote sensing, high performance computing, and process-based modeling of SOC dynamics. Combined together, these modern tools were essential for reducing error between model estimates and field observations. When standardized by the range in size of the SOC stocks projected, our observed RMSE between estimates and projections of SOC stocks is consistent with respective measures of error from digital soil mapping methods used at comparable scales^17,38–40^. Below, we discuss in greater detail the implications of combining parameter optimization and uncertainty analysis with high-resolution forcing data for future applications, as well as the unique information provided by high resolution modeling of SOC dynamics across complex landscapes.

### Parameter estimation and uncertainty

Our estimates of SOC stocks across the RCEW-CZO suggest that the scales over which measurements are taken, and for which models are parameterized, influence the predicted sensitivity of SOC stocks to different environmental control factors. Differences between our optimized parameters and the default parameter estimates reflect the improvements made by calibrating the model to fit local observations (Fig. 2). Our findings also underscore important differences in parameter estimates generated by when calibrating MIMICS at local- versus continental-scales (Fig. 3, 4). For example, results from our model parameterization suggest a higher temperature sensitivity of microbial catabolism in our optimized parameterization of MIMICS across local gradients than was derived from the default parameterized generated from site level means across a continental-scale gradient (Table 1, Fig. 3)^26^. This parallels findings from Bradford et al.^24^ who found strong scale dependence on the strength of causative relationships between environmental drivers and decomposition rates, suggesting that accounting for local heterogeneity may be important to consider for inferring proximal controls over SOC turnover that are used in model parameterizations^41^.

**Table 1:** MIMICS parameters included in the Monte Carlo (MC) optimization and the subsequent best-fit values found for the Reynolds Creek Experimental Watershed and Critical Zone Observatory (RCEW-CZO) calibration data.

Parameter	Description	Default prior^a^	Best-fit scaling factor^b^	Scaling factor proposal range^c^	Units
*V*_slope_	Decomposition max rate coefficient	0.063	3.85 ± 0.8	0.4–4	ln(mg Cs (mg MIC)^−1^ h^−1^) °C^−1^
*V*_int_	Decomposition max rate coefficient	5.47	1.16 ± 0.4	0.3–3	ln(mg Cs (mg MIC)^−1^ h^−1^)
*K*_slope_	Decomposition half saturation coefficient	0.017, 0.027, 0.017	0.84 ± 1.0	0.4–4	ln(mg C cm^−3^) °C^−1^
*K*_int_	Decomposition half saturation coefficient	3.19	1.55 ± 0.7	0.3–3	ln(mg C cm^−3^)
*τ*	Microbial biomass turnover rate	0.30 × e^1.3(*f*clay)^, 0.20 × e^0.8(*f*clay)^	0.46 ± 0.5	0.3–3	h^−1^
*MGE*	Microbial growth efficiency	0.55, 0.25, 0.75, 0.35	0.54 ± 0.3	0.2–2	mg mg^−1^
*D*	Desorption rate from SOMp to SOMa	1.5 × 10^–5^ × e^-1.5(fclay)^	0.08 ± 0.03	0.001–0.3	h^−1^
*f*_*p*_	Fraction of τ partitioned to SOMp	0.015 × e^1.3(fclay)^, 0.01 × e^0.8(fclay)^	0.34 ± 0.3	0.01–4	–

^a^Further information regarding MIMICS parameters and default initial values provided in^26^

^b^Parameter proposals in the MC algorithm were conveyed as a factor of the initial prior value(s). Presented standard deviation is derived from the parameter ensemble (n = 30) with RMSE 1.8–2.0

^c^Factor proposals for each parameter were confined to agree with natural analogs and to reduce computational demands.

See Methods for further details.

Results from our parameter ensemble have higher temperature sensitivity for microbial kinetics and lower MGE than the initial parameterization (Fig. 3). These parametric changes related to microbial physiology underscore how microbial communities can influence the rate of organic matter turnover through their catabolic capacity, as well as the fate of SOM via microbial anabolism^42^. Interestingly, our parameter ensemble did not indicate any statistical correlation between parameter combinations related to microbial catabolism or anabolism (Fig. 4), yet MIMICS represents a theoretical expectation that tradeoffs between microbial growth rates and growth efficiency should be linked in the parameterization of copiotrophic and oligotrophic communities^43,44^. Ultimately, the parameter estimates from our ensemble result in faster turnover and lower retention of fresh organic matter inputs– findings consistent with conclusions of Shi et al.^45^. The parameter ensemble also produced much longer turnover times for physicochemically protected soil C pools, correcting known biases in MIMICS^22,46^. Turnover times of SOM_p_ pools, however, still have large uncertainty, leading to large variation in the size of this soil C pool (Figs. 3, 4). Moving forward, we expect information about soil C fractions and their radiocarbon ages will provide an important additional constraint to reduce these uncertainties.

The parametric uncertainty analysis also identified what additional observations could help constrain model representations of microbial physiology and SOC persistence (Figs. 3, 4). Specifically, observations of litter decomposition rates, the relative size of SOC pools (e.g., from density fractionation), laboratory measurements of microbial carbon use efficiency (or microbial growth efficiency, MGE), and the turnover rate for mineral associated organic matter (from radiocarbon measurements on mineral associated organic matter) are directly relatable to the model and can be used as constraints during parameter optimization to better calibrate the model parameters. Auto-correlations between model parameters suggest that data pertaining to any one property may help to constrain other parameters in the model. For example, desorption rates (D) and the fraction of microbial turnover that is physicochemically stabilized (f_p_) show high correlation with each other (Fig. 3). This suggests that radiocarbon measurements that provide a constraint on mineral protected SOM turnover would reduce uncertainty ranges for both of these parameters. Similarly, measurements of microbial growth efficiency are becoming more common, and may also help constrain the parameterization of microbial turnover in the model. We note, however, that laboratory assays of MGE are short-term measurements of (near) instantaneous growth efficiency that are dependent on substrate quality and particulars of the chosen methodological approach^47,48^. As such, the absolute values of MGE from any incubation study may not be directly representative of these fixed parameters that are currently used in MIMICS, highlighting the importance of improving our understanding and representation of this key parametric uncertainty in soil biogeochemical models^47,49–51^. More relevant, may be developing an understanding of how microbial community composition reflects environmental gradients and may influence variation in MGE over space and time. Overall, this work illustrates improved capabilities and opportunities to exchange information between process-based models, field measurements, and laboratory observations. Such exchanges are critical for ensuring that model representations of SOC dynamics align with natural systems and produce accurate projections.

### High resolution inputs, SOC stocks, and projections

Our study highlights the data challenges associated with trying to generate measurements that are needed for this kind of work. Even at a well-studied site like the RCEW-CZO that has a wealth of soils data, co-located measurements of additional driver data that are needed to parameterize MIMICS were largely absent (e.g. soil properties, soil temperature and moisture, plant productivity, and litter chemistry). Thus, we derived these from remote sensing and statistical modeling. Although these methods are still imprecise, they better account for local heterogeneity in biotic and abiotic factors known to influence SOM persistence and mark an important step forward in considering how we can parameterize and validate soil biogeochemical models.

Process-based models of SOC dynamics employ varied constructs and environmental properties to predict SOC^52^. The MIMICS model has been shown to have a relatively high dependence on plant productivity, as opposed to greater dependence on soil texture found in other, similar process-based models of SOC dynamics^7^. In practice, our high-resolution mapping application benefited from MIMICS dependence on NPP, since estimates of productivity are readily derived from high resolution multispectral imagery (< 30 m^2^)^53,54^. Methods for estimating vegetative properties from multispectral imagery are broadly effective for most environments^55–57^. In contrast, relatively few methods exist for collecting information on soil properties (e.g. clay content) at similar resolutions^58^. The reduced influence of clay content relative to NPP on SOC dynamics in MIMICS has been proposed as a potential cause for observed discrepancies between SOC stocks simulated by MIMICS relative to field observations and current theory^7^. However, results from our parameter optimization procedure demonstrate that such discrepancies in MIMICS projections of protected SOC can be rectified through the model parameterization (Fig. 4D).

Surprisingly, we found that the inclusion of soil moisture in the MIMICS model, a factor with strong influence on SOC stocks at global scales^59^, did not improve estimate accuracy across the RCEW-CZO watershed. This result may in part be explained by the fact that soil water availability along the elevational gradient in the RCEW-CZO is strongly correlated with productivity estimates from remote sensing at the site^60,61^. Thus, the spatially distributed productivity estimates that we developed likely captured the effects of moisture and was a significant factor explaining SOC stocks. Alternatively, accuracy of the spatial soil moisture data available may not have been sufficient to appropriately convey soil moisture conditions at the spatial resolution of this study, and ongoing work at the watershed is focused on generating high spatial and temporal resolution estimates for the site. We see such efforts as critical for estimating temporal variation in heterotrophic respiration fluxes and improving projections of soil C responses to climate change. In contrast, we found that spatially continuous mean annual soil temperature (MAST) derived from soil temperature observations, elevation and estimated incoming solar radiation was a significant factor explaining SOC stocks. Seyfried et al.^62^ and more recently Seyfried et al.^63^ documented pronounced aspect effects on local soil temperature at the RCEW-CZO that rival larger elevational gradients. For example, differences in soil temperature associated with aspects in small catchments of the RCEW-CZO (< 2 km^2^) are greater than differences in MAST observed along a 900 m elevation gradient (5.5 versus 4.4 °C), exemplifying the importance of using fine scales to model environmental processes in complex landscapes.

Our assessment of SOC stocks and sensitivities at high spatial resolution across the RCEW-CZO provides a robust example of how fine scale applications of process-based models of SOC dynamics can provide useful information to support land practice and policy decisions. High spatial resolution estimates of SOC stocks across the entire RCEW-CZO provide insight into landscape variation in SOC stocks that may be expected across the semi-arid environments of the Great Basin, USA (Fig. 5). We see this providing opportunity for land managers to better plan land use and focus valuable resources towards specific areas where the protection or management of SOC is warranted. The model also makes testable predictions about the size of particular soil C pools and their change over environmental gradients (Fig. 6), that could be validated with additional measurements taken at field relevant scales. Further, our idealized environmental change scenarios demonstrate the ability to assess SOC stability across a diverse landscape (Fig. 7).

The idealized scenarios used here illustrate the potential to use a calibrated model like MIMICS to make spatially-explicit projections of SOC responses to environmental change. Rangelands exist over ~ 40% of the Earth’s ice-free land and account for ~ 30% of terrestrial SOC to a depth of 1 m^64^. The global potential for rangeland C sequestration has been estimated to range from 0.3 to as much as 1.6 Pg CO_2_-eq per year^65^. Moreover, experiments like these can provide estimates of the magnitude of soil C change, and their associated uncertainty, that can be expected over large areas given incremental changes in local conditions (like temperature, productivity, and their interactions). High resolution process-based modeling of soil C dynamics provides a critical tool for evaluating such potential, as well as for identifying, protecting and harnessing sequestration potential in soil across earth’s surface.

## Conclusion

In this study, we show that accurate, high resolution (10 m^2^) projections of 0–30 cm SOC stocks may be generated by combining remotely sensed environmental data and process-based modeling, with the aid of parameter optimization methods and high-performance computing. Leveraging the power of modern parameter optimization techniques and machine learning to improve Earth systems models remains a widespread area of emphasis across disciplines in environmental science^66,67^. The Monte Carlo simulation used for parameterizing the MIMICS model provided the cornerstone for this study, allowing us to calibrate model parameters, quantify the impacts of parametric uncertainty, and generate spatially explicit estimates of soil C stocks with high-resolution environmental data. For future studies and applications, we see great promise for similar approaches to assist with model parameterization and spatial extrapolations of SOC projections.

Required adjustments to the MIMICS parameters controlling SOC distributions and environmental sensitivity in simulations for the RCEW-CZO support a deepening discussion in ecology and Earth system science regarding the potential fallacies of inference when scales between observations and the environmental processes being studied are misaligned^24^. Observed differences in the best-fit parameters between fine and continental scale applications of the MIMICS model also support other recent studies in demonstrating the substantial variability found in soil processes at fine spatial scales^11,68,69^. The demonstrated ability to perform fine scale simulations of SOC dynamics thus provides a critical tool and resource for linking environmental observations with current theory and associated model representations. Such ability is critical for improved assessments and understanding of landscape drivers of SOC persistence, and for the projection of SOC response to changes in environmental conditions.

Moving forward, the proven approach and developed algorithms from this study provide a strong foundation for continued model improvement and further high-resolution applications. Our analysis of the parametric uncertainty in the model projections shows the direct opportunities for additional observational data pertaining to microbial properties and SOC persistence to further refine MIMICS parameter estimates. Further potential also exists to generate more complex, fine scale estimates and projections of SOC dynamics by adapting the methods from this study to similarly enhance the spatial application scale and parameterization of the CN-coupled^70^ and soil depth resolved^71^ versions of the MIMICS model.

## Methods

### Study area

The Reynolds Creek Experimental Watershed and Critical Zone Observatory (RCEW-CZO) in southwestern Idaho, USA covers 239 km^2^, with 13 km^2^ of active cropland near the valley bottom. The RCEW-CZO is situated in a mountainous region and contains a large elevational gradient (~ 1100–2100 m) that gives rise to diverse climates, soils and vegetation throughout the watershed^36,63^. Vegetative productivity is severely limited by low water availability in lower elevations (< 1400 m) of the watershed, which receive an average of 230–300 mm of precipitation each year. Mean annual temperature across the lower elevations of the watershed is 9.1 °C. In the lowland regions, the vegetation is predominately sagebrush (*Artemisia spp.*) with a sparse underlying mix of grasses and forbs. At the mid-elevations of the watershed (1400–1700 m), pockets of aspen (*Populus tremuloides*) and willow (*Salix spp.*) begin to emerge in riparian areas, as well as on some of the northern facing slopes, where winter snowpacks supply sufficient water. At higher elevations, Juniper (*Juniperus spp.*) and Douglas fir (*Pseudotsuga menziesii*) stands are common, though not pervasive, supported by far greater annual precipitation and lower mean annual air temperatures, averaging 350–994 mm per year and 5.4–8.5 °C. Soils across the RCEW-CZO watershed are mixed and predominantly originate from either felsic material (i.e., granitic Idaho Batholith^72^) or mafic basaltic flows^73^. Soils are generally deep across the RCEW-CZO (> 1 m not including colluvium)^74^. Soil development is typically greatest at higher elevation in convergent topography. For the top 30 cm of soil, the mafic derived soils are generally loams (average sand, silt, clay: 41%, 39%, and 20%), while the felsic are generally sandy loams (average sand, silt, clay: 59%, 25%, and 16%^16,75^). Across the RCEW-CZO, approximately 85% of the soils are classified as Mollisols, with another 13% classified as Aridisols^76^.

### Field observations of SOC stocks

Soil C stocks were previously determined at unique locations across the RCEW between 2014–2016^75,77,78^. For model calibration we used previously collected data pertaining to total soil C stocks from 0–30 cm at 89 locations across the RCEW-CZO. The median 0–30 cm soil C stock was used where soil data overlapped within a 10 m^2^ area.

### The MIMICS model and parameter optimization methods

The MIcrobial-Mineral Carbon Stabilization (MIMICS) model has been widely used for estimation of soil C across diverse ecosystems and has been found to perform well across ecosystem gradients and at global scales^22,26,43,46,70,79^. Here we use a version of the model that calculates equilibrium SOC stocks based on the mean annual values for net primary productivity, soil temperature, litter lignin:N ratio and the soil clay content. Soil moisture may also be used as a control on soil C dynamics in the MIMICS model^22^. However, model testing using soil moisture inputs derived from aerial imagery and a snow model did not improve model accuracy of SOC stocks estimates at RCEW, and thus soil moisture was not included as a model input. We assumed all field observations of SOC included in the study are at steady state and ignored the interannual and seasonal dynamics of climate and vegetation. In kind, all model simulations were carried out to steady state. These assumptions present uncertainty and limitations regarding the insights and projections from the model, but it remains common practice to allow for best use of available data^43,46,79^.

The flow of C through the MIMICS model is controlled by a number of rate parameters (Table 1, Fig. 1)^26^. These parameters control the simulated turnover and persistence of litter, microbial biomass, and soil C pools (Fig. 1; Methods). Model parameters broadly fit into three categories related to microbial catabolic capacity (decomposition kinetics described by V_max_ and K_es_), microbial anabolism (microbial growth efficiency and turnover; MGE and *τ,* respectively), and physicochemical protection of SOC (Fig. 1).

Initially, we used a Hamiltonian Markov Chain Monte Carlo (MCMC) algorithm^80^ to determine the best fit values for eight of the MIMICS model parameters with the strongest control over litter, microbial biomass, and soil C pools. Uniform prior distributions for the MCMC were given a mean and standard deviation equal to the parameter values provided by Wieder et al.^26^. In practice, the best-fit parameterizations determined over multiple MCMC runs were not consistent, suggesting that substantial equifinality exists in the model construct. To account for and gain further insight into how equifinality in model parameterization impacts model estimates, we proceeded to perform a Monte Carlo (MC) simulation involving 500,000 MIMICS model runs with unique, random parameter combinations. To improve performance of the MC simulation, we proceeded to tune the minimum and maximum limits on each of the uniform parameter proposal distributions (i.e. the scaling factor proposal range, see Table 1) to ensure that the range encompassed all values with the potential to produce viable model outcomes (see criteria below). Specifically, using repeated trials of the MC simulation, the scaling factor proposal range was extended when viable model outcomes were observed within 10% of the proposal value limit Conversely, scaling factor ranges were narrowed when parameter proposals above or below a certain limit resulted in implausible model outcomes (i.e., Desorption rates (D) > 0.3 produce protected SOM pool (SOMp) turnover rates below the constraint of 50 years (See further model outcome constraint details below)). Ultimately, all parameterizations from the MC simulation yielding a RMSE between field and estimated bulk SOC values of < 2.0 kgC m^-2^ were included in the parameter ensemble used throughout the study. Further, a parameter set was only deemed viable if it passed a set of binary filters to confirm that the parameterization produced plausible values for microbial C (1–3% of the total SOC), the relative abundance of microbial r- vs. K-strategist (0.1–10), litter C stocks (10–40% of total SOC), and the turnover time of C in the protected SOM pool (50–400 years). For brevity, we refer to these accurate (RMSE < 2.0) and plausible parameterizations throughout the manuscript as the parameter ensemble (n = 30). The lowest RMSE observed over all combinations was 1.72 kgC m^-2^.

Previous studies have optimized MIMICS parameters using MCMC^79^ and shuffled complex evolution (SCE) algorithms^46^. We chose to employ a MC simulation to optimize the model parameters due to the simplicity of the algorithm relative the MCMC and SCE methods. The MC simulation requires no statistical assumptions and has no inherent potential to bias the optimized parameters chosen. The MC optimization approach is computationally inefficient compared with MCMC and SCE, which required us to use high performance computing clusters to complete the necessary number of model simulations (n = 500,000). We performed a threefold cross-validation to ensure the MC optimization method results were not biased by the nature of the RCEW forcing data or the number of simulations performed. Each cross-validation of the MC optimization procedure used a unique sub-sample (n = 60) of the field observations of SOC stocks to derive the optimized parameter distributions. Cross-validation sub-samples were stratified by co-located estimates of GEP to ensure consistency in the scope of the model projections. The resulting optimized parameter distributions from cross-validation simulations showed no concerning disparities between the subsample distributions and the distributions derived from the full dataset (Figure SI3).

We used the following approach to quantify the variability in MIMICS C pool estimates produced by the unique parameterizations in the parameter ensemble. For each location in the dataset, we calculated the range of estimated values and then divided that by the minimum estimated value. We then calculated the average and standard deviation of those location specific estimate variability factors across all locations.

### Spatial data analysis

Model estimation of soil C stocks across the entirety of the RCEW-CZO required continuous spatial data layers for elevation and incoming solar radiation, soil temperature, net primary productivity, soil clay content and the lignin:N ratio for the dominant vegetative source of surface litter. Digital elevation and soil clay content layers were used as provided online courtesy of the U.S. Geological Survey (https://earthexplorer.usgs.gov/) and USDA Web of Soil Survey^76^ respectively. Clay content was procured from the Web Soil Survey as an average value for the soil layer from 0–30 cm. The underlying source of the WSS clay data is the SSURGO database^81^. To create a spatial map of lignin:N content of the dominant vegetative species across the RCEW, the vegetation map provided in Seyfried et al.^82^ was paired with corresponding litter lignin:N values reported in the literature. From the digital elevation map, incoming solar radiation was calculated using the ESRI Solar Radiation Toolset, which calculates net incoming solar radiation by simulating the solar path over the digital elevation map surface while accounting for atmospheric effects, site latitude and elevation, slope, aspect, daily shifts of the sun angle, and effects of shadows cast by surrounding topography^83^.

#### Soil temperature

A continuous spatial layer for mean annual soil temperature (MAST) was created based on the relationship found between soil temperature, elevation and incoming solar radiation. Across the full extent of the elevation gradient of Reynolds Creek, and for soils with similar aspects, there is a strong relationship between soil temperature and elevation. However, soil temperatures differ substantially for soils with opposing north and south facing aspects, as shown by Godsey et al.^84^ and Seyfried et al. ^62,63^. To compensate for these aspect effects on MAST, estimated annual incoming solar radiation was used as an adjustment factor as shown in the following equation:

$$MAST = - 0.0087(ELEV) + 22.43 - (solarMID - solarIN/tMOD)$$

where ELEV represents elevation (m); solarMID represents the average incoming solar radiation across the RCEW-CZO (553,833 W m^-2^); solarIN represents the incoming solar radiation at the estimate location (W m^-2^) and tMOD is employed as a scaling factor (46,385.8 W m^-2^). All coefficients, and the tMOD scaling factor, were determined using a linear optimization algorithm set to minimize error between observed and estimated MAST values for a series of locations across the RCEW-CZO (n = 35, data from Seyfried et al.^62,63^ and Godsey et al.^84^). The spatially dispersed and depth stratified MAST observation data showed no significant differences in MAST across soil depth increments of 5, 10, 20 and 30 cm. Based on this evidence, all MAST data available for soil depths < 30 cm were used to calibrate the MAST estimation equation. Correlation between observed and predicted MAST values equaled R^2^ = 0.73 (Fig. SI4). The resulting continuous spatial map of MAST produced is assumed to be representative of MAST from 0–30 cm soil depth.

#### Gross primary productivity

Hyperspectral satellite-based imagery was used to generate a continuous spatial map of gross ecosystem productivity (GEP) across the RCEW-CZO. Following work previously completed by Fellows et al.^60^, Landsat 5 imagery courtesy of the U.S. Geological Survey was collected across 10 years, from 2002 to 2011. From this data, specific images were chosen from each year to determine the maximum modified soil-adjusted vegetation index (MSAVI2) values at a 30 m^2^ resolution across the RCEW-CZO. The MSAVI2 index has been demonstrated as a viable method for identifying differences in vegetative productivity^85–87^. MSAVI2 was calculated using the following equation:

$$MSAVI2 = \left( {2*NIR + 1 - \sqrt {\left( {(2*NIR + 1)^{2} - 8*(NIR - RED)} \right)} } \right) / 2$$

where NIR is the near infrared band reflectance and RED is the red band reflectance value. Generation of a continuous map of GEP was then based on the observed relationship between maximum mean annual MSAVI2 and observed measures of GEP across the RCEW-CZO (GEP = 1972.8(MSAVI2) + 101; r = 0.69). Observations of GEP were also collected between 2002–2011 and were generated by eddy covariance at three elevation stratified flux towers. The relationship between observed and predicted GEP is shown in (Fig. SI4). To run the MIMICS model, which requires a measure of vegetative productivity in the form of annual net primary productivity, we estimated ANPP to equal half of GEP.

### Spatial extrapolation and environmental change scenarios

As needed, all spatial data layers were resampled into raster format with 10 m^2^ resolution using nearest neighbor resampling method, and all layers were re-projected to a common projection and coordinate reference system (World Geodetic System (WGS) 1984, Universal Transverse Mercator (UTM) Zone 11). For model calibration and validation datasets, pixel values from the spatial data layers for GEP, MAST, clay content and litter lignin:N content were extracted at the coordinates of sampling points.

We performed MIMICS simulations for each grid cell in the RCEW-CZO spatial data (2.4 million grid cells). Each grid cell simulation involved producing estimates from each member of the parameter ensemble (n = 30). The average estimate at each grid cell was then used to construct the continuous maps (Figs. 5, 6, 7). The corresponding parametric uncertainty maps were similarly generated by calculating the standard deviation of the estimates for each grid cell. Continuous spatial estimates of SOC responses to idealized scenarios were similarly performed. Idealized scenarios were applied through a uniform shift in the corresponding forcing data layer (+ 10% increase in GPP or + 1 °C MAST) prior to the model simulations. The High-Performance Computing (HPC) resources provided by Idaho State University and the U.S. Department of Energy Idaho National Laboratory were invaluable for the high-resolution spatial projections, allowing us to complete over 300 million MIMICS simulations.

### Statistical analysis and code

All modeling, analysis and spatial mapping for this study were performed in R^88^. Root mean square error and correlation between model estimates and field data were specifically performed using the Metrics (https://github.com/mfrasco/Metrics) and base R packages respectively^88^. The MIMICS model and the developed Markov Chain Monte Carlo and Monte Carlo simulation algorithms were composed in R programming scripts and are available in open-access repositories on GitHub (https://github.com/piersond/MIMICS_HiRes, https://github.com/wwieder/MIMICS/tree/sandbox). The MIMICS_HiRes repository provides all modeling and analysis code associated with this study, as well as useful startup information and simplified examples to assist future use. Scripts in the repository were assembled specifically for use with HPC to reduce the time required for model parameterization and simulations.

## Supplementary Information

Supplementary Information.

## Data Availability

The spatial and tabular datasets used for model forcing, calibration and validation within this study are publicly available from Boise State University ScholarWorks. (https://scholarworks.boisestate.edu/reynoldscreek/26/; https://doi.org/10.18122/reynoldscreek.26.boisestate).

## Code availability

Codes used for MIMICS parameterizations, estimates, projections and spatial extrapolations associated with this manuscript are publicly available on GitHub. (https://github.com/piersond/MIMICS_HiRes; https://doi.org/10.5281/zenodo.5794655).
