# Supplementary material for: Optimizing process-based models to predict current and future soil organic carbon stocks at high-resolution
Source: Sci Rep. 2022 Jun 25;12:10824. doi: 10.1038/s41598-022-14224-8 (PMC9233666; doi:10.1038/s41598-022-14224-8)
Supplement: Supplementary file 1 — Supplementary Information. [file 41598_2022_14224_MOESM1_ESM.docx]

**Optimizing process-based models to predict current and future soil organic carbon stocks at high-resolution**

Derek Pierson^1*^, Kathleen A. Lohse^1,2^, William R. Wieder^3,4^, Nicholas R. Patton^2,5^, Jeremy Facer^1^, Marie-Anne de Graaff^6^_,_ Katerina Georgiou^7^, Mark S. Seyfried^8^, Gerald Flerchinger^8^, Ryan Will^9^

^1^ Department of Biological Sciences, Idaho State University, Pocatello, ID, USA

^2^ Department of Geosciences, Idaho State University, Pocatello, ID, USA

^3^ Climate and Global Dynamics Laboratory, National Center for Atmospheric Research, Boulder, CO, USA

^4^ Institute of Arctic and Alpine Research, University of Colorado, Boulder, CO, USA

^5^ School of Earth and Environment, University of Canterbury, Christchurch, NZ

^6^ Department of Biological Sciences, Boise State University, Boise, ID, USA

^7^ Physical and Life Sciences Directorate, Lawrence Livermore National Laboratory, Livermore, CA, USA

^8^ Agricultural Research Service, Northwest Watershed Research Center, Boise, ID, USA

^9^ Department of Geosciences, Boise State University, Boise, ID, USA

# SUPPLEMENTARY INFORMATION

| **Table SI1.** Cross validation estimate statistics. | | | |
| --- | --- | --- | --- |
| **Ensemble (Line color in Fig. SI3)** | **Bias** | **Correlation** | **Root mean square error (kg C m^-2^)** |
| Complete dataset (Grey) | 0.99 ± 0.14 | 0.79 ± 0.01 | 1.85 ± 0.07 |
| Cross Validation Sample 1 (Green) | 1.63 ± 0.28 | 0.75 ± 0.04 | 1.98 ± 0.08 |
| Cross Validation Sample 2 (Blue) | 1.12 ± 0.14 | 0.78 ± 0.02 | 1.87 ± 0.07 |
| Cross Validation Sample 3 (Red) | 1.11 ± 0.16 | 0.79 ± 0.01 | 1.87 ± 0.10 |
| See Figure SI3 for distributions of parameter values in each ensemble | | |  |
| Values reported as ensemble mean ± standard deviation | |  |  |


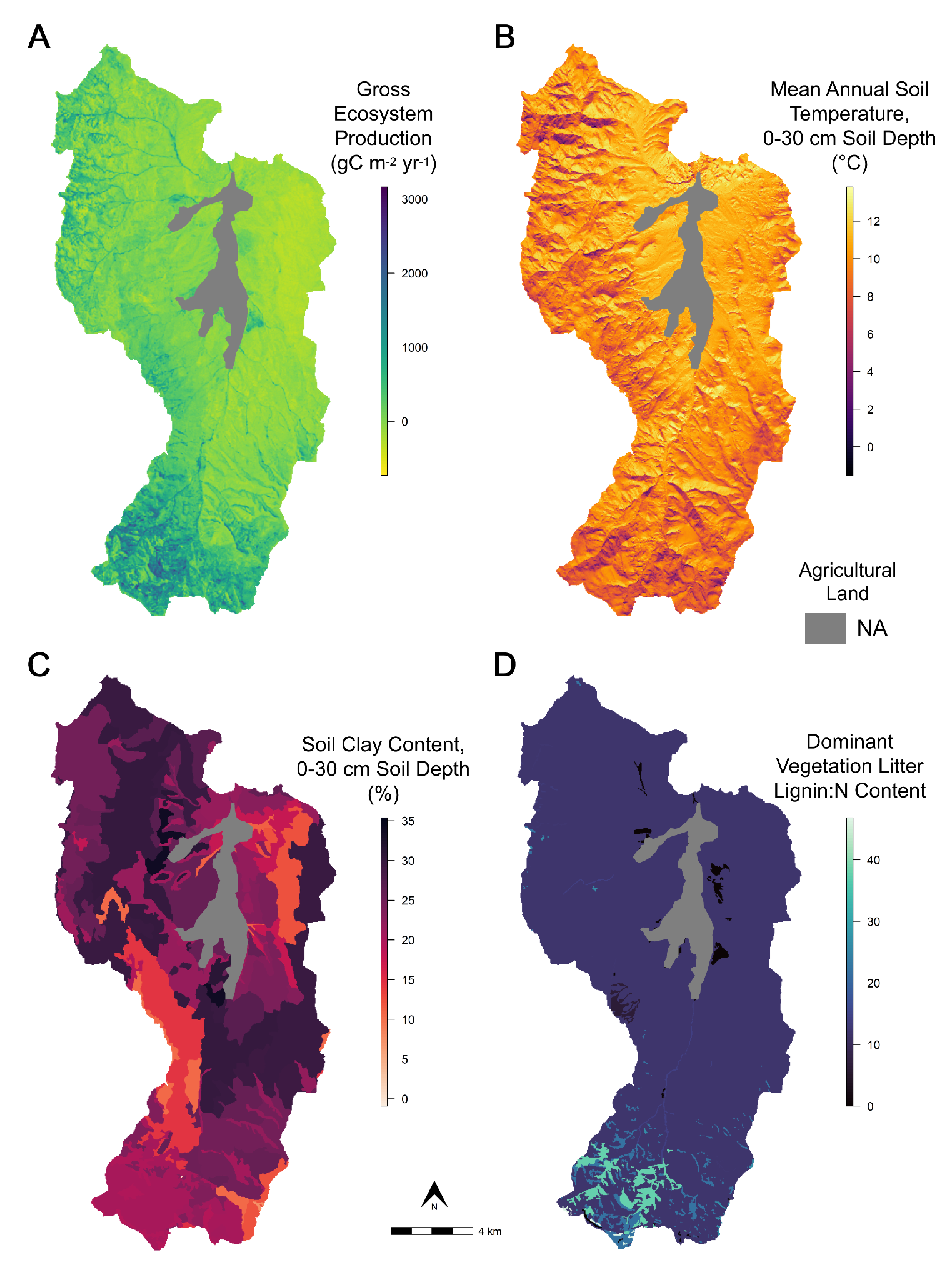


**Figure SI1.** Forcing data used for model projections of soil organic carbon across the Reynolds Creek Experimental Watershed and Critical Zone Observatory, including A) gross ecosystem productivity, B) mean annual soil temperature, C) soil clay content and D) dominant vegetation litter lignin:N content. (Generated by free software R, https://www.R-project.org/).

**
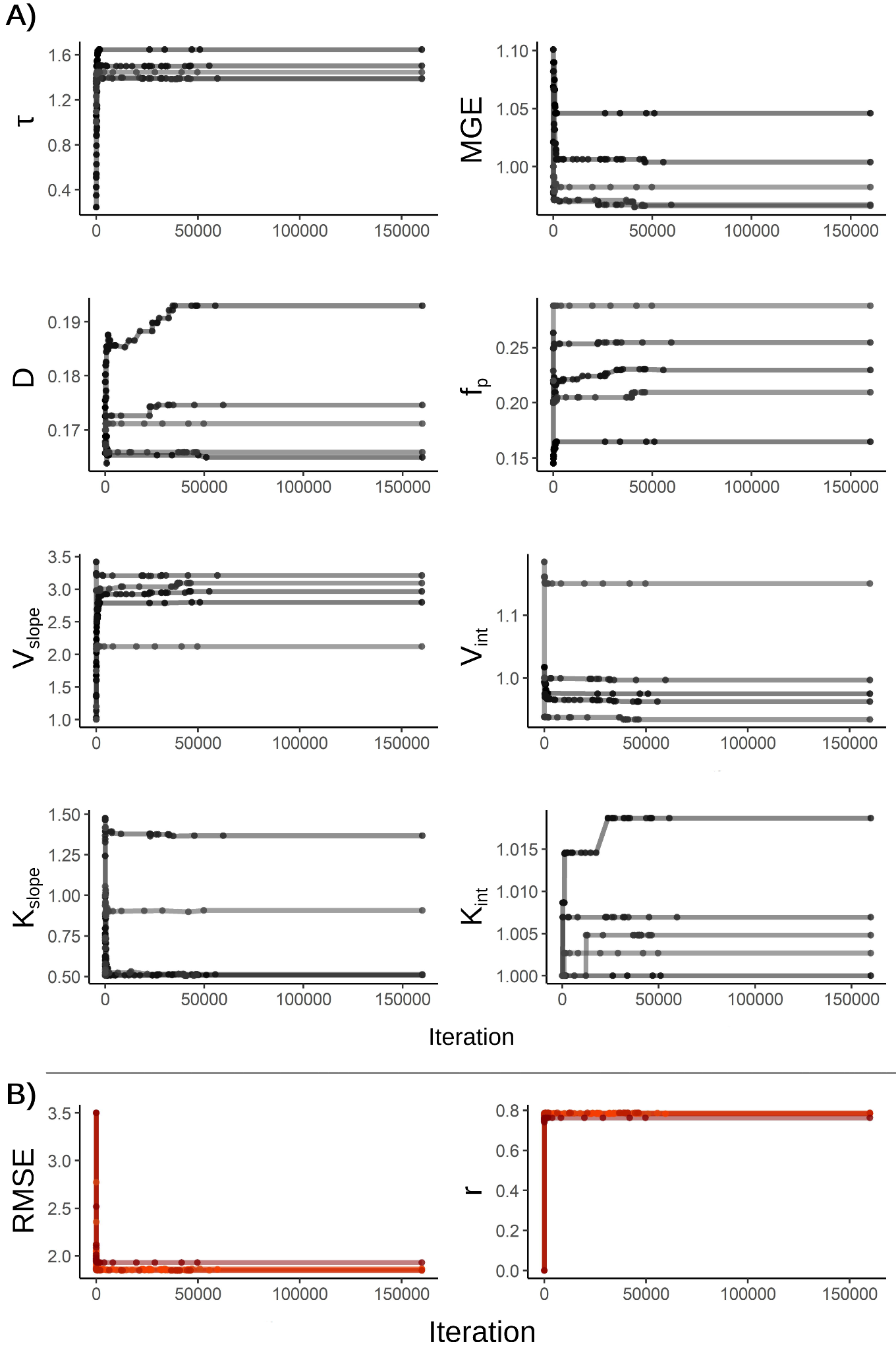
**

**Figure SI2.** A) Parameter values found from repeated applications of the developed Markov Chain Monte Carlo (MCMC) algorithm used to determine the best-fit parameters for the MIMICS model. Parameter values are displayed as a factor of the default parameter values for MIMICS provided by Wieder et al. 2015. B) Corresponding correlation and root mean square error (RMSE) between the field observations and model estimates of SOC stocks used to calibrate the model. (Generated by free software R, https://www.R-project.org/).

**
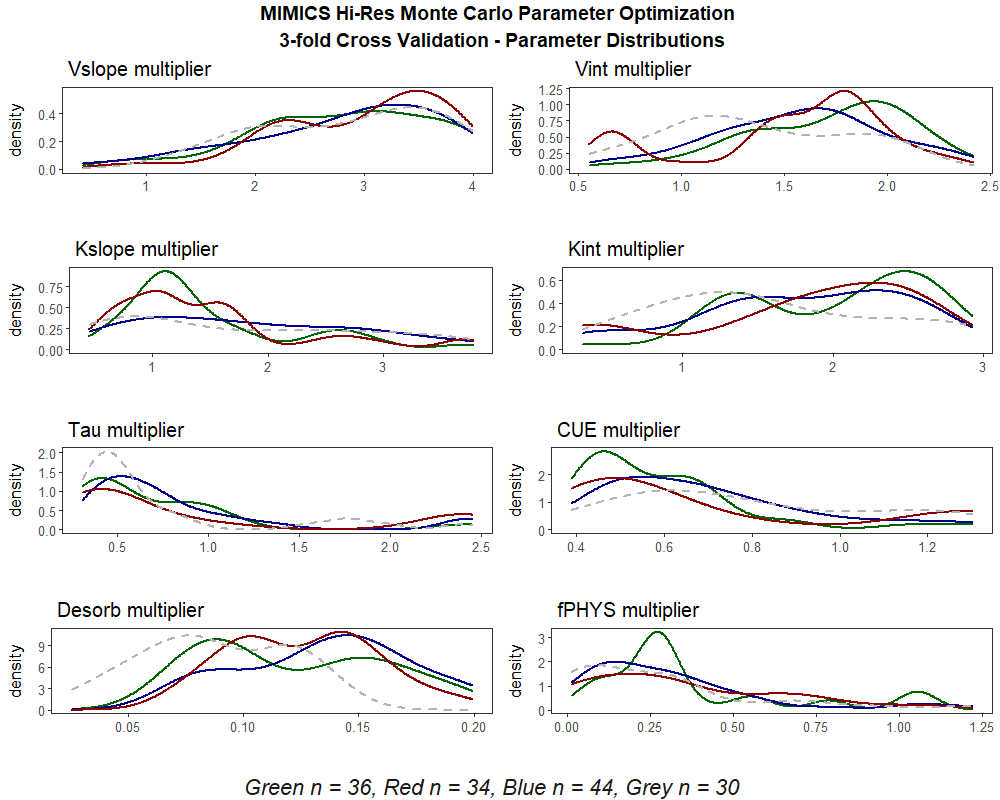
**

**Figure SI3.** Distributions for MIMICS parameter scaling factors determined by Monte Carlo simulation based on agreement (RMSE < 2) between simulated and observed soil organic carbon stocks across the Reynolds Creek Experimental Watershed and Critical Zone Observatory. Dashed line represents the optimized parameter distribution obtained when calibrating with the complete dataset (n=89) of field observations of SOC stocks. Colored lines represent the optimized parameter value distributions obtained for three unique, sub-sampled datasets of field SOC stocks (n=60). (Generated by free software R, https://www.R-project.org/).

#
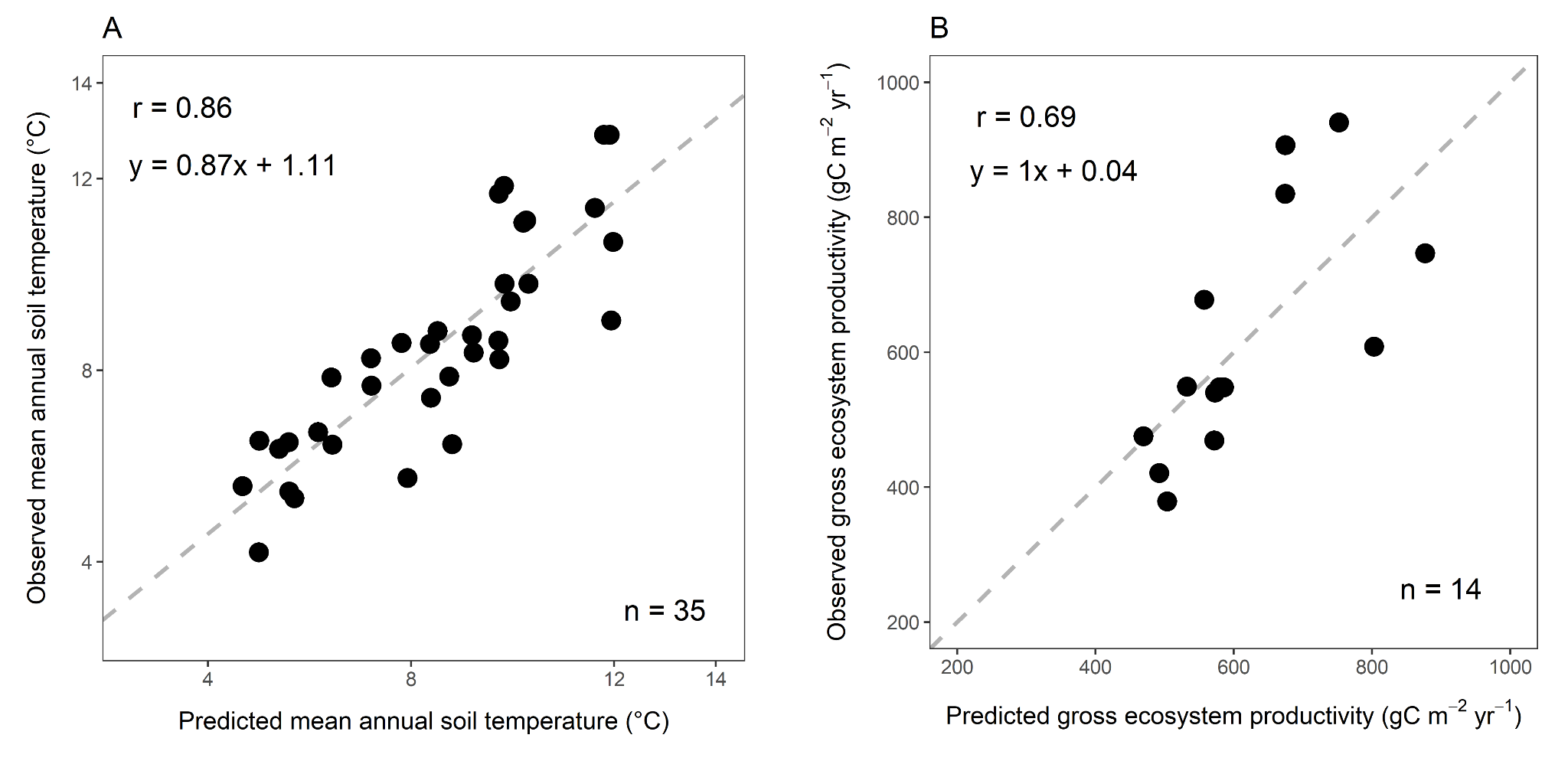


**Figure SI4.** Predicted versus observed (a) mean annual soil temperature and (b) gross ecosystem productivity at locations across the Reynolds Creek Experimental Watershed and Critical Zone Observatory. (Generated by free software R, https://www.R-project.org/).
